# Overexpression of Ent-Kaurene Synthase Genes Enhances Gibberellic Acid Biosynthesis and Improves Salt Tolerance in *Anoectochilus roxburghii* (Wall.) Lindl.

**DOI:** 10.3390/genes16080914

**Published:** 2025-07-30

**Authors:** Lin Yang, Fuai Sun, Shanyan Zhao, Hangying Zhang, Haoqiang Yu, Juncheng Zhang, Chunyan Yang

**Affiliations:** 1 Medical Plant Exploitation and Utilization Engineering Research Center Fujian, Provincial Key Laboratory of Resources and Environment Monitoring & Sustainable Management and Utilization, Sanming University, Sanming 365004, China; yangl@fjsmu.edu.cn (L.Y.); 13859688205@163.com (S.Z.); 18005985018@163.com (H.Z.); 2 Maize Research Institute, Sichuan Agricultural University, Chengdu 611130, China; faisun@ucdavis.edu (F.S.); yhq1801@sicau.edu.cn (H.Y.); 3 Guizhou Institute of Prataculture, Guiyang 550006, China

**Keywords:** *ArKS*, GAs, salt stress, *A. roxburghi*

## Abstract

**Background**: *Anoectochilus roxburghii* (Wall.) Lindl. (*A. roxburghii*) was widely used in traditional Chinese medicine and also as a health food in China. Gibberellins (GAs) are plant hormones that regulate various aspects of growth and development in *A. roxburghii*. Ent-kaurene synthase (KS) plays a crucial role in the biosynthesis of GAs in plants. However, there is limited functional analysis of KS in GA biosynthesis and its effect on salt tolerance, especially in *A. roxburghii*. **Methods**: The *ArKS* genes were cloned from *A. roxburghii*, and its salt tolerance characteristics were verified by prokaryotic expression. Under salt stress, analyze the regulation of *KS* gene on GA and active ingredient content by qRT-PCR and HPLC-MS/MS, and explore the mechanism of exogenous GAs promoting active ingredient enrichment by regulating the expression level of the *KS* under salt stress. **Results**: The *ArKS* protein was highly homologous to KSs with other plant species; subcellular localization of KS protein was lacking kytic vacuole. The transformants displayed a significant increase in salt tolerance under the stress conditions of 300 mM NaCl. And the expression of *ArKS* genes and the GAs accumulation was downregulated under the salt stress; among them, the contents of GA3, GA7, GA8, GA24, and GA34 showed a significant decrease. It was further found that there was an increase (1.36 times) in MDA content and a decrease (0.84 times) in relative chlorophyll content under the salt conditions from *A. roxburghii.* However, the content of active constituents was elevated from *A. roxburghii* under the NaCl stress, including polysaccharides, total flavonoids, and free amino acids, which increased by 1.14, 1.23, and 1.44 times, respectively. Interestingly, the *ArKS* gene expression and the chlorophyll content was increased, MDA content showed a decrease from 2.02 μmoL·g^−1^ to 1.74 μmoL·g^−1^ after exogenous addition of GAs, and the elevation of active constituents of polysaccharides, total flavonoids, and free amino acids were increased by 1.02, 1.09, and 1.05 times, implying that GAs depletion mitigated the damage caused by adversity to *A. roxburghii*. **Conclusions**: The *ArKS* gene cloned from *A. roxburghii* improved the salt tolerance of plants under salt stress by regulating GA content. Also, GAs not only alleviate salt tolerance but also play a key role in the synthesis of active components in *A. roxburghii*. The functions of *KS* genes and GAs were identified to provide ideas for improving the salt tolerance and quality of ingredients in artificial cultivation from *A. roxburghii*.

## 1. Introduction

Gibberellins (GAs), a class of diterpene phytohormones commonly found in advanced plant species, are crucial in regulating tissue growth and development [1]. They played a crucial role in various processes, including the germination of seeds, the elongation of stems, the expansion of leaves, and the development of flowers [1]. These compounds of GAs are known to improve adaptability under biotic stresses, such as pathogen attacks, and abiotic stresses, such as UV irradiation [2,3]. The biosynthesis of GAs begins with the conversion of geranylgeranyl diphosphate (GGDP) into diterpenoid cyclic hydrocarbon intermediates, which is catalyzed by ent-copylyl diphosphate synthase (CPS) and ent-kaurene synthase (KS), yielding ent-kaurene, the precursor of GAs [4]. KS was the last key rate-limiting enzyme that forms the precursor of GAs [5]. The enzymes of KS, crucial for the GA biosynthetic pathway, have been successfully identified and characterized in plant systems [6,7,8].

The enzymes of KS and their encoding genes have been successfully identified and characterized in many other plants, such as *Cucurbita maxima* Duchesne ex Lam.(*C. maxima*), *Camellia sinensis* cv. Baihaozao (*C. sinensis*), *Scoparia dulcis* L. (*S. dulcis*), *Oryza rufipogon* Griff. (*O. rufipogon*), and *Picea glauca* (Moench) Voss (*P. glauca*) [4,9,10,11,12]. The *KS* gene of *C. sinensis* exhibited high expression in tender stems and roots, as well as many other tissues that are rapidly growing. This tissue-specific expression suggests the role of this phytohormone in providing precursors necessary for regulating tissue growth and development [4]. Compared with the wild type, the expression level of the *KS* gene and the content of gibberellin in the mutant cabbage were lower than those in the wild type [13].

In rice, the expression of the *KS* genes was found to be significantly decreased in response to salinity stress [14,15]. It is thought that the GA precursor encoded by the *KS* genes gives rise to diterpenoids and other hormones such as strigolactones, which are involved in root development or regulating stomata under salt stress [16]. In addition, altering GA content by overexpressing or knocking out the KS genes was found to affect secondary growth and secondary regeneration of vascular tissue in poplar [17]. The inhibition of cambium regeneration from the mutant poplar could be partially restored by GA application, and the mutant cabbage phenotype could be restored to that of the wild type after exogenous GA treatment [13,17].

*Anoectochilus roxburghii* (Wall.) Lindl. (*A. roxburghii*), a leaf-based economic crop from Orchid, is recognized for its medicinal and food properties, particularly in the treatment of cancer and allergies (Figure S1). This species is known to contain various active ingredients relating to plant quality and taste, such as polysaccharides, kinsenoside, flavonoids, and free amino acids [18,19]. Due to its protected status, propagation of *A. roxburghii* typically occurs through artificial cultivation or tissue culture to facilitate its rapid multiplication [20,21]. The salinity stress has been found to influence plant growth and accumulation of active ingredients in cultivated *A. roxburghii* [22,23]. However, it is limited to the functional analysis of KS in GA biosynthesis and its effect on the enrichment of active ingredients and stress tolerance in *A. roxburghii*.

In this research, the *KS* gene was cloned from *A. roxburghii*. Its function of salinity tolerance was elucidated by subcellular localization, measurement of relative physiological indicators, such as content of GAs, chlorophyll, malondialdehyde (MDA), and active ingredients, in response to salinity stress and exogenous addition of Gas, as well as ectopic transformation in *Escherichia coli*. The heterologous expression and content of GAs of the transformants in response to salinity stress were detected by real-time quantitative PCR (RT-qPCR) and Liquid Chromatography-Tandem Mass Spectrometry (LC-MS/MS), respectively. The results highlighted the crucial role that the *ArKS* genes play in the biosynthesis of GAs and salinity tolerance of *A. roxburghii*.

## 2. Materials and Methods

### 2.1. Sample Preparation

The *A. roxburghii* seedlings were disinfected with 10% NaClO solution for 5 min. The stem nodes with buds were cut and transplanted onto Murashige and Skoog (MS) plates, incubated in an environmental chamber with a photoperiod of 12 h light (3000 Lux)/12 h of dark, a temperature of 28 °C, and a relative humidity of 60–80%. After four months, the emerging seedlings were transferred to plastic mesh grids and aquacultured in Hoagland nutrient solution. All the *A. roxburghii* seedlings were divided into the CK group and the LS group, with the only difference being that 100 mM NaCl was added to the nutrient solution of the LS group. After 15 d, 50 nM GA_3_ and 50 nM GA_8_ were added for salinity stress and exogenous phytohormone treatment, respectively. Three biological replicates were designed for each of the above treatments.

The whole plants of each replicate were sampled at different time points at 0 (control), 0.5, 1, 2, 4, 8, 12, 24 h (1 d), 15 d (before adding GA_3_ and GA_8_), and 20 d after NaCl treatment. After a quick freeze in liquid nitrogen, total RNA was isolated by using the Qiagen RNeasy plant mini kit (Qiagen, Hangzhou, China) and reverse transcribed into cDNA by using the PrimeScript RT reagent Kit (TaKaRa, Dalian, China).

### 2.2. Gene Cloning

According to the RNA sequencing (RNA-seq) annotations, a specific pair of primers (5′-ATGGTCCCATCTCCAAGATTT-3′/5′-TTAAGCATCAGAAAGGACGTGG-3′) was meticulously crafted and used to amplify the open reading frame (ORF) of the *ArKS* gene from the cDNA sample of the 0 h control within PrimeSTAR HS DNA Polymerase (TaKaRa, Dalian, China). The annealing temperature was 62 °C, and other amplification processes were referred to the PCR amplification process. The amplified product was then purified by using the Universal DNA Purification Kit (Tiangen, Beijing, China), appended with dATP ends by using TaKaRa TaqTM (Takara, Dalian, China), and cloned into the pMD19-T vector (TaKaRa, Dalian, China) [21].

### 2.3. Bioinformatic Analysis

The constructed vector was sequenced at Sangon Biotech (Shanghai, China) Co., Ltd. corporation and aligned against the NCBI platform to clarify its gene structure. Subsequently, it was analyzed for the physical and chemical properties, secondary structures, functional domains, genetic frameworks of the predicted proteins, subcellular localization, and phylogenetic tree by using different bioinformatic tool such as ProtParam, GOR IV, TMHMM Server v. 2.0, SWISS-MODEL, and WoLF PSORT [21].

### 2.4. Vector Construction

A pair of homologous recombination primers (5′-catttggagaggacagggtacccgggATGGTCCCATCTCCAAGATTT-3′/5′-tcgcccttgctcaccatggtactagtAGCATCAGAAAGGACGTGG-3′) was designed and used to amplify the ORF of the *ArKS* gene without the stop codon. The amplified products were cloned into expression vector pCAMBIA2300 by using the CloneExpress One Step Cloning Kit (Vazyme, Nanjing, China) to create the fusion expression vector of the *ArKS* genes and the enhanced green fluorescent protein gene (*eGFP*), termed *ArKS-eGFP* (see Figure S2).

Another pair of homologous recombination primers (5′-cgggatccATGGTCCCATCTCCAAGATTT-3′/5′-ccaagcttTTAAGCATCAGAAAGGACGTGG-3′) was carefully crafted to incorporate recognition sequences (indicated by the lowercase letters in parentheses) to enhance the effective amplification of the ORF of *ArKS*, which does not possess a termination codon. The amplified product was then cloned into the pET-28a(+) plasmid to generate expression vectors for expressing the *ArKS* protein (see Figure S3) using a CloneExpress One Step Cloning Kit (Vazyme, Nanjing, China).

### 2.5. Subcellular Localization

According to the methods reported in our previous study [21], the 35S-*KS-eGFP* plasmids were attached to gold particles, with the empty 35S-*eGFP* vector serving as a control. Onion bulbs were bombarded with a helium biolistic gun (Bio-Rad, USA) and incubated for another 24 h in the dark at 28 °C. The localization of the *ArKS* protein within subcellular compartments was assessed by utilizing GFP as a reporter. Onion epidermal cells were infiltrated with bacterial cells containing KS-GFP plasmids. The fluorescence signal was observed using a confocal microscope (Olympus BX63, Toyko, Japan).

### 2.6. Prokaryotic Expression

The pET-28a(+)-KS plasmid was incorporated into the *E. coli* Rosetta (DE3) strain, with a control group utilizing an empty prokaryotic pET-28a(+) plasmid. Following transformation, positive colonies were identified by PCR amplification and used for isopropyl β-D-thiogalactopyranoside (IPTG) with 1 mM induction for 4 h [23]. Each culture was then diluted to an OD_600_ of 0.6 and used for two experiments.

One experiment was solid-state culture: in accordance with the methodologies outlined, the cultures were 1:10 serially diluted to 1:10^4^ [24,25]. The microliters from each dilution were plated onto ampicillin LB agar plates containing 300 mM NaCl. After 16 h of incubation, colony growth was assessed.

Another experiment was liquid culture: cell growth with the vector pET-28a(+)—KS under 150, 200, 250, 300, and 350 mM NaCl was monitored by measuring the absorbance at OD_600_ [8].

### 2.7. RT-qPCR

A specific primer pair (5′-GATAGAATCCACGAGGGCCG-3′/5′-ACTGCACCGACCATTTCCTT-3′) was designed to amplify a 172 bp fragment of the *ArKS* gene. In addition, a 221 bp fragment of the *Actin2* gene was amplified using primer pairs (5′-CGGGCATTCACGAGACCAC-3′/5′-AATAGACCCTCCAATCCAGACACT-3′) and used as an internal control [21]. A two-step temperature cycle was conducted by using ChamQ Universal SYBR qPCR Master Mix (Vazyme, Nanjing, China) in the CFX96TM Real Time System (Bio-Rad, Hercules, CA, USA). The relative expression levels were calculated and normalized, as well as analyzed for statistical significance, by using the 2^−ΔΔCT^ method of the CFX Manger™ software v 2.0 (Bio-Rad, Hercules, CA, USA) [21].

### 2.8. Quantification GAs

The samples of whole plants sampled at 0 and 15 days of the salinity stress were frozen using liquid nitrogen and pulverized into a fine powder. A total of 50 mg of the powder was subjected to extraction utilizing 10 μm of an internal standard (10 ng/mL). The extracts were agitated at 4 °C for 15 min, followed by centrifugation at 12,000 r/min for 10 min to isolate the supernatant. The supernatant was then concentrated overnight using a concentration device until desiccated. Following this, it was vortexed for 10 min, subjected to another centrifugation at 12,000 r/min at 4 °C for 5 min, and 800 μL of C_4_H_8_O_2_ from the upper phase was transferred into a brown injection vial. This extraction procedure was reiterated, and the resultant extracts were amalgamated with the initial extraction solution. The consolidated extracts were then reconstituted in 100 μL of a 90 acetonitrile/10 water (*v*/*v*) solution and filtered through a 0.22 μM filter membrane.

Ten microliters of each filtrate were introduced into an LC-MS/MS (QTRAP^®^ 6500+) that was fitted with an ACQUITY UPLC CSH C18 column (1.7 μm, 2.1 × 100 nm). A gradient elution strategy was implemented, commencing with a mobile phase composition of 95% H_2_O with 0.05% HCOOH (A) and 5% HCOOH with 0.05% CH_3_CN (B) for an initial duration of 0 min. This was followed by a transition to 5% A and 95% B over the course of 10 min, reverting back to 95% A and 5% B for 1 min, then to 5% A and 95% B for 0.1 min, and ultimately returning to 95% A and 5% B for 3.9 min, all operated at a flow rate of 0.35 mL/min and a column temperature set at 40 °C. The MS/MS parameters included an electrospray ionization source temperature maintained at 550 °C, a mass spectrometry voltage of 5500 V/-4500 V in positive/negative ion mode, and a curtain gas pressure of 35 psi. Each ion pair underwent analysis through scanning of the declustering potential and collision energy.

### 2.9. Measurement of Related Physiological Indicators

The leaves of *A. roxburghii* were assayed for relative chlorophyll content using SPAD-502 Plus (Konica, Tokyo, Japan) at the same time each day at 0 and 15 days, which represents the photosynthetic efficiency and growth of *A. roxburghii*. GA_3_ and GA_8_ were added exogenously on the fifteenth day, and the relative chlorophyll content of *A. roxburghii* leaves continued to be assayed on the twentieth day using SPAD-502 Plus (Konica, Tokyo, Japan).

Using trichloroacetic acid (TCA) and thiobarbituric acid (TBA), the content of malondialdehyde was determined by spectrophotometry [22].

The samples collected at 0, 15, and 20 days post-induction were subjected to a drying process at 55 °C and subsequently pulverized into a fine powder. A precise 0.1 g of this powder was then extracted in 10 mL of distilled water utilizing an ultrasonic device, conducted at a temperature of 80 °C for a duration of 3 h. Following extraction, the resulting solutions underwent centrifugation at 5000 rpm for 20 min, after which they were adjusted to a final volume of 10 mL. The determination of polysaccharide concentration was executed via the phenol-sulfuric acid assay, employing glucose as the calibration standard, with absorbance readings taken at 490 nm using a UV-1800 spectrophotometer (Shimadzu, Kyoto, Japan). The quantitative assessment of polysaccharide content was thereafter computed accordingly.

The samples collected at 0 and 15 days of induction with NaCl and 20 days of induction with NaCl adding GA_3_ and GA_8_ were subjected to a drying process at 55 °C subsequently ground into a fine powder. A 0.1 g portion of this powder underwent extraction in 95% ethanol utilizing an ultrasonic device at 25 °C for a duration of 30 min. Determination of total flavonoid content using the Al(NO_3_)_3_ colorimetric detection method [22].

Each sample, including the control (0 days) and those from 15 and 20 days of induction, was pulverized with 10% CH_3_COOH, and the volume was brought up to 100 mL. The supernatants obtained were then extracted, and the amino acid content was determined using the ninhydrin method, with an amino acid solution as the standard, for absorbency at 570 nm.

## 3. Results

### 3.1. Open Reading Frame and Putative Proteins

Complementary DNA samples from *A. roxburghii* were employed for the amplification of segments exceeding 2000 bp, utilizing primers designed based on the information provided (see Figure S2). The *ArKS* features a solitary open reading frame comprising 2169 bp, encoding a protein of 723 amino acids with a molecular mass of 82.182 kDa, an isoelectric point of 6.36, and a GRAVY index of −0.107, with the molecular formula being C3690 H5793 N977 O1072 S37. The subcellular localization was predicted to be cytoplasm. The protein’s secondary structure encompasses 42.19% α-helices, 13.42% β-strands (extended strands), and 44.40% random coils.

### 3.2. Multiple Sequence Alignment

**Figure 1:**
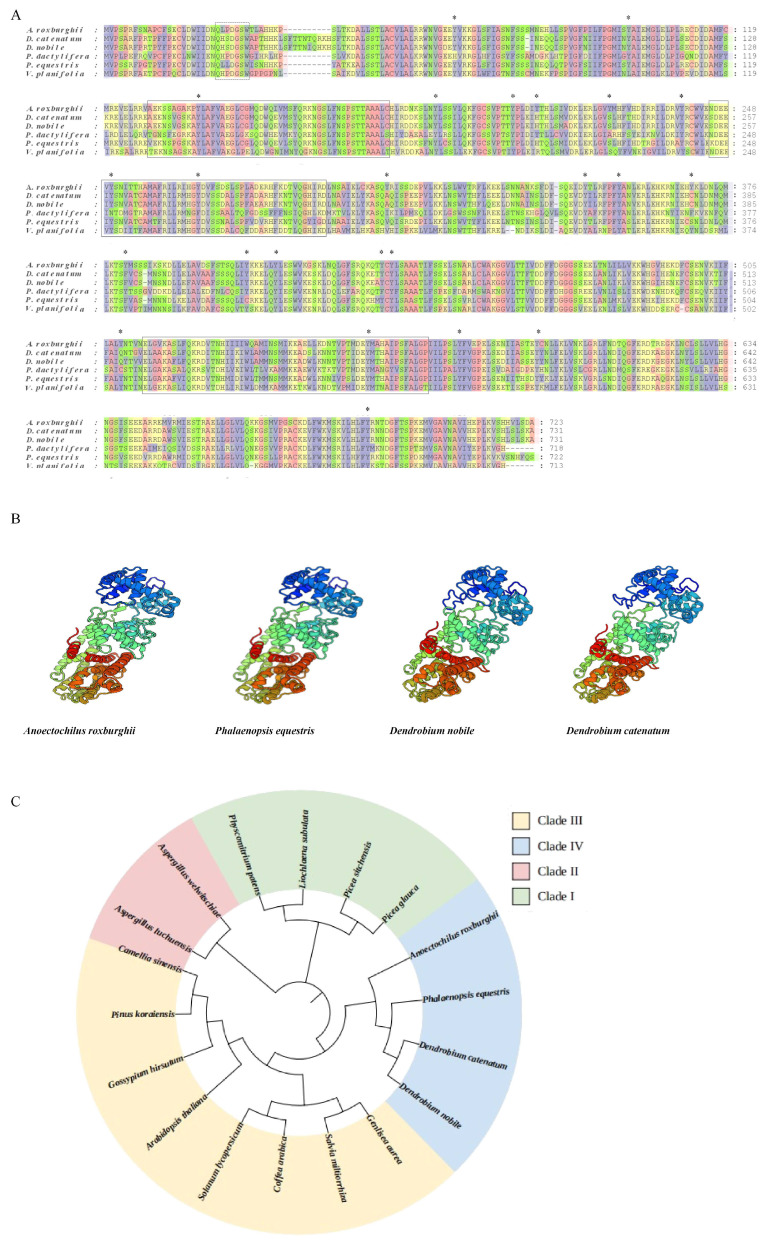
Molecular characterization of the *ArKS* gene from bioinformatics. (**A**): The functional domain structure of the ArKS protein and KS proteins in other plants. Solid boxes denote conserved motifs, dashed boxes indicate QXXDGSW motifs, and ‘*’ represent the phosphorylation sites. (**B**): Three-dimensional structural models of the *ArKS*, *DcKS*, *DnKS*, and *PeKS*. (**C**): Phylogenetic tree of *A. roxburghii* putative proteins and functional KS proteins deposited in other plants.

Three conserved domains were identified at positions 139–188, 194–242, and 523–572, which are essential for the protein’s significant function. In addition, the motif QXXDGSW, conserved in plant diterpene cyclases, was contained within *ArKS*. Furthermore, 24 phosphorylation sites were identified in the *ArKS* protein (Figure 1A). These findings imply that the isolated cDNA is responsible for encoding a KS-type diterpene cyclase. The three-dimensional structural models of *ArKS*, *PeKS*, *DnKS*, and *DcKS* exhibited high similarity (Figure 1B). The presumed *ArKS* protein was grouped into the same subgroup as the confirmed functional KS proteins of *Dendrobium nobile* Lindl. (*D. nobile*, *DnKS*), *Dendrobium catenatum* Lindl. (*D. catenatum*, *DcKS*), *A. roxburghii*, and *Phalaenopsis equestris* (Schauer) Rchb.f. (*P. equestris*, *PeKS*), substantiating that the *ArKS* belongs to the KS protein family (Figure 1C).

### 3.3. Subcellular Localization of KS Proteins

**Figure 2:**
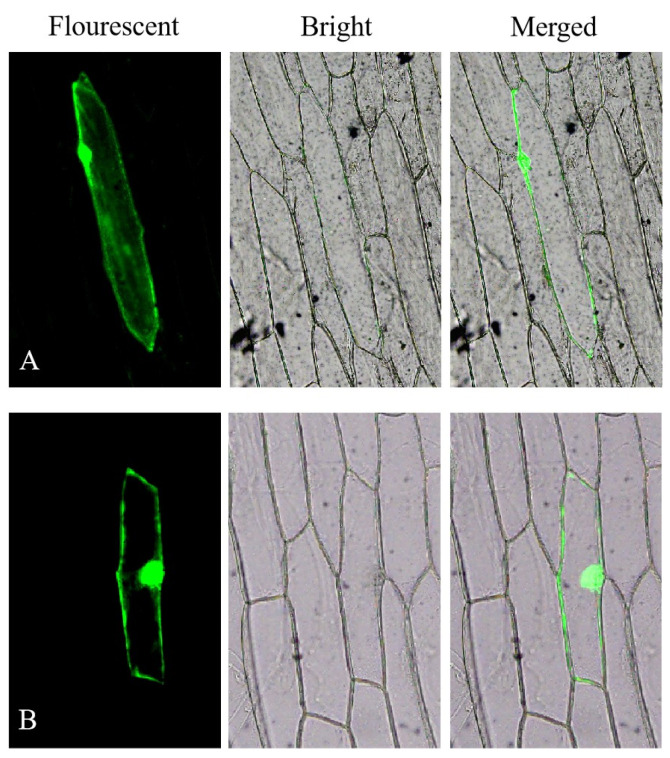
Subcellular localization of the KS protein. (**A**): epidermal cells of onion transformed with pC2300-35S-eGFP (expression of fusion gene eGFP under the regulation of the CaMV 35S promoter); (**B**): epidermal cells of onion transformed with pC2300-35S-KS-eGFP (expression of fusion gene KS-eGFP under the regulation of the CaMV 35S promoter) derived from *A. roxburghii*.

Confocal microscopy images revealed that the GFP fusion protein was distributed widely throughout the epidermal cell of onions (Figure 2A). And the KS-GFP fusion protein lacked a kytic vacuole (Figure 2B). This finding does not conflict with the bioinformatics predictions regarding the localization of the KS protein in the cytoplasm and nucleus.

### 3.4. Overexpression of KS Gene

**Figure 3:**
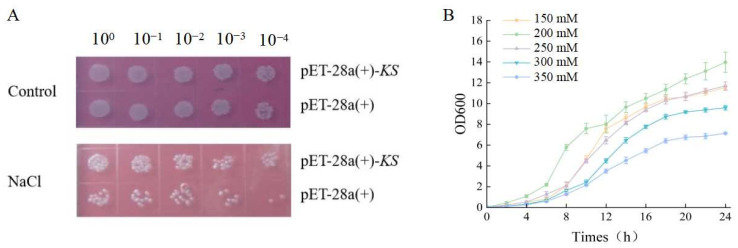
Growth characteristics of *E. coli* transformed with pET-28a(+)-*KS* under NaCl. (**A**): Growth characteristics of *E. coli* colonies transformed with pET-28a(+) and pET-28a(+)-*KS*. (**B**): The growth of *E. coli* transformed with pET-28a(+)-*KS* under different NaCl.

The colony counts on LB agar plates decreased significantly as the dilution fold increased. Furthermore, there was a significant difference between colonies transformed with the vector pET-28a(+)-KS and those transformed with the empty vector pET-28b(+). When subjected to 300 mM NaCl stress, the *E. coli* colonies carrying pET-28a (+)-KS demonstrated considerably greater resistance to salt stress compared to those with pET-28a (+) (Figure 3A). In addition, the growth of cells with the vector pET-28a (+)—KS was affected by different salt solubility. At 200 mM NaCl, cell growth was optimal, with higher salt solubility leading to slower cell growth. However, at lower NaCl concentrations (150 mM), cell growth was also affected (Figure 3B).

### 3.5. Relative Expression Levels and GA Accumulation Under Salinity Stress

**Figure 4:**
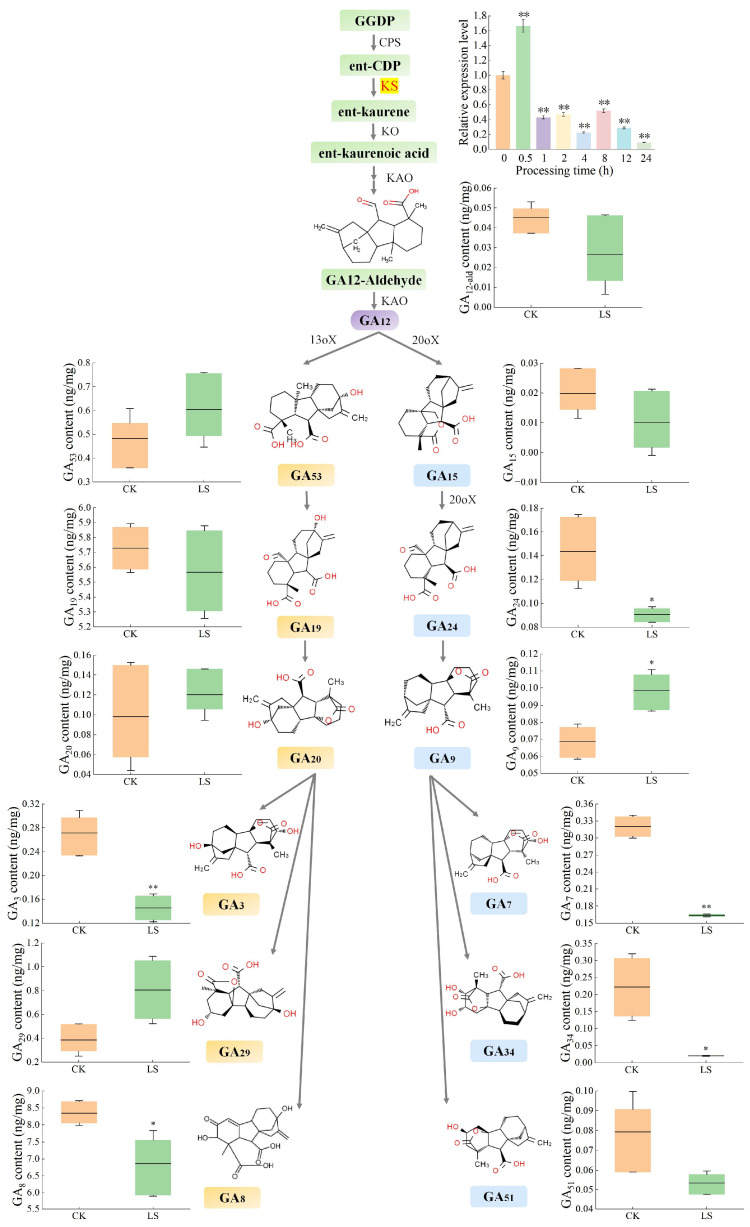
Levels of GAs in *A. roxburghii* under normal conditions, as well as under NaCl stress. CK refers to non-inducing conditions; LS indicates NaCl stress after 15 days. ‘*’ and ‘**’ indicate significance compared to the control at the 0.05 and 0.01 levels, respectively.

In response to the salinity stress, the relative expression levels of the *ArKS* genes were notably increased, reaching a peak value of 1.48 times at 0.5 h of 100 mM NaCl stress treatment. Subsequently, these levels declined drastically, resulting in only one-tenth of its highest peak at the 24th h of the treatment and even fewer than the negative control (the 0 h control, Figure 4).

**Figure 5:**
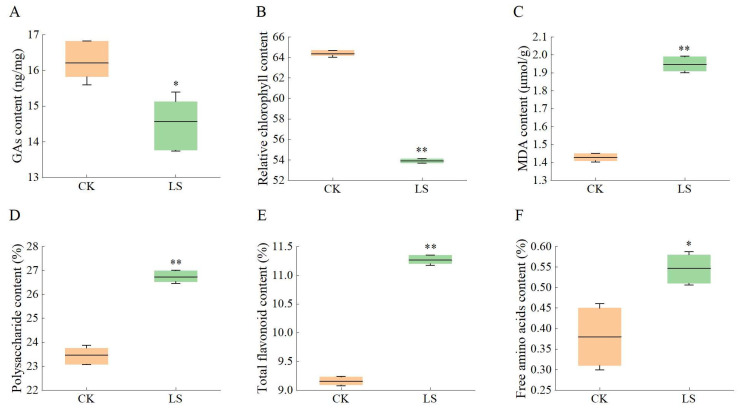
The GAs, relative chlorophyll, MDA, and active ingredient contents under non-inducing conditions and salt stress from *A. roxburghii*. (**A**): The contents of GAs; (**B**): The relative chlorophyll content; (**C**): The contents of MDA; (**D**): The contents of polysaccharide. (**E**): The contents of total flavonoids. (**F**): The contents of free amino acids. The orange box is for the non-inducing condition; the green box stands for the NaCl stress. ‘*’ and ‘**’ indicate significance compared to the control at the 0.05 and 0.01 levels, respectively.

The total amount of GAs was 16.21 ng·mg^−1^, in the non-stress conditions (the 0 h control) in the whole plant (Figure 5A). Among them, the accumulation of GA_12_ was 0.05 ng/mg. The content of GA_24_, GA_51_, GA_9_, GA_7_, and GA_34_ was 0.14, 0.08, 0.07, 0.28, and 0.22 ng·mg^−1^, respectively, which were downstream products of GA_15_ (0.02 ng·mg^−1^). Other metabolic products were GA_53_, GA_19_, GA_20_, GA_29_, GA_3_, and GA_8_, the accumulation of which was 0.48, 5.71, 0.10, 0.39, 0.27, and 8.35 ng·mg^−1^, respectively. The accumulation of GA_8_ was the highest of all 13 kinds of GAs from *A. roxburghii*, and GA_15_ was the lowest, only 0.02 ng·mg^−1^. Except for GA_19_ and GA_8_, the contents of other GAs were not exceeding 1 ng·mg^−1^. The accumulation of GA_19_ and GA_8_ was much higher than that of other GA_S_, which came from the same branch (Figure 4). In response to NaCl induction, the total amount of GAs decreased to 14.56 ng·mg^−1^ (Figure 5A). Among them, the accumulation of GA_51_, GA_7_, GA_34_, and GA_3_ was decreased by 0.05, 0.16, 0.02, and 0.15 ng·mg^−1^ (*p* < 0.01), and G_24_ and G_8_ decreased by 0.09 and 6.86 ng·mg^−1^ (*p* < 0.05). However, the accumulation of GA_9_ and GA_29_ showed upregulation under the low salt induction, indicating 1.44 (*p* < 0.01) and 2.09 (*p* < 0.05) times. The expression levels of GA_12_, GA_15_, GA_53_, GA_19_, and GA_20_ remained unchanged under the NaCl stress (Figure 4).

### 3.6. Response of Related Physiological Indicators to Salinity Stress

**Figure 6:**
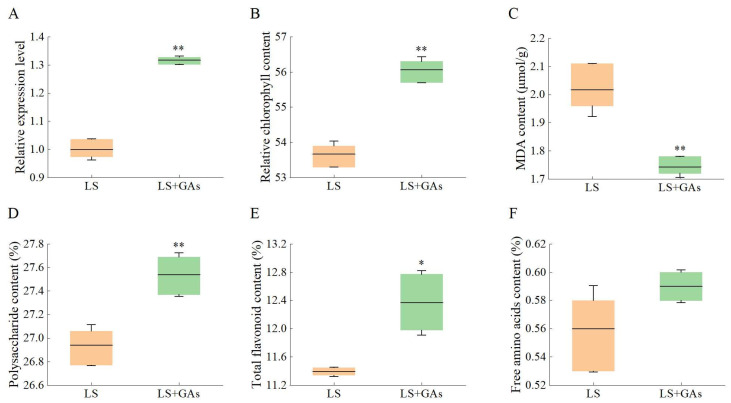
The relative expression levels of *KS*, relative chlorophyll, MDA, and active ingredient content under salt stress with exogenous GA compensation from *A. roxburghii*. (**A**): The relative expression levels of *KS*; (**B**): The relative chlorophyll content; (**C**): The contents of MDA; (**D**): The contents of polysaccharide; (**E**): The contents of total flavonoids; (**F**): The contents of free amino acid. The orange box is for the NaCl stress; the green box stands for the NaCl stress with GAs. ‘*’ and ‘**’ indicate significance compared to the control at the 0.05 and 0.01 levels, respectively.

The total amount of GAs was 16.21 ng·mg^−1^ under non-stress conditions (the 0 h control). Correspondingly, under salinity stress, the total amount of GAs decreased to 14.56 ng·mg^−1^ (Figure 5A). In the CK group, the relative chlorophyll content remained stable (64.37) at the 0th day. However, relative chlorophyll content gradually decreased after NaCl treatments. The relative chlorophyll content at the 15th day was 83.74% of the untreated (Figure 6B). The initial accumulation of MDA in the 0th d control from *A. roxburghii* was measured at 1.43 μmoL/g. This value saw a significant increase due to NaCl induction, reaching 1.95 μmoL/g by the 15th day, with the upregulation of expression corresponding to 1.36 times that of the 0 h control (Figure 5C). And treated with salt stress, the active ingredient accumulation was increased, with varying degrees observed among different factors. When untreated, polysaccharide content was 23.47%. During the 15-day stress period, the levels of polysaccharide content rose by 1.14 (26.73%) times when exposed to NaCl (Figure 5D). The total flavonoid accumulation continued to rise, particularly with an increase of 1.23 times (from 9.16% to 11.26%) under NaCl treatment at the 15th day (Figure 6E). In *A. roxburghii*, the untreated levels of free amino acids were 0.38%. When treated, the contents were also increased under NaCl salinity stress, reaching 1.44 times by the fifteenth day (Figure 6F).

### 3.7. Relative Expression Levels of KS, Relative Chlorophyll, MDA, and Active Ingredient Content Under Salt Stress with Exogenous GAs Compensation

The relative expression of *KS* genes after GA addition was significantly higher than the relative expression without addition under NaCl conditions. Relative expression levels of the *KS* gene were 1.31 times higher than those without GAs (Figure 6A). When treated with NaCl stress, the relative chlorophyll content was shown to be elevated at 53.67 from the *A. roxburghii*. After exogenous addition of the phytohormone GAs, the relative chlorophyll content was increased to 56.07, and the stress of NaCl was partially counteracted (Figure 6B). The initial accumulation of MDA was measured at 2.02 μmoL/g on the 20th d under NaCl stress from *A. roxburghii*. This value saw a significant decrease due to NaCl induction, reaching 1.74 μmoL/g by the 20th day, with the regulation of expression corresponding to 0.86 times (Figure 6C). The polysaccharide content was 26.94% under the NaCl stress and GAs rose to 27.54% on the 20th day (Figure 6D). The total flavonoid accumulation continued to rise, particularly with an increase of 11.39% under NaCl treatment, and treated with GAs, the total flavonoid accumulation increased, with varying degrees observed among different factors (Figure 6E). And the contents of free amino acids were increased from 0.56% to 0.59% by GAs treated under the NaCl stress (Figure 6F).

## 4. Discussion

GAs were hormones found in plants that regulate various aspects of growth and development, such as seed germination, stem growth, leaf expansion, and seed maturation [26,27]. The KS enzyme was crucial in the biosynthesis of GAs, and the *KS* gene was also important for plants’ tolerance to abiotic stresses [4,10]. In this research, the *KS* gene was isolated from the cDNA of *A. roxburghii*. The similarities between the ORF and predicted protein sequences were analyzed in comparison to the known functional KS proteins from *D. catenatum* and *P. equestris*. The KS proteins exhibited three conserved domains: the QXXDGSW motifs, the aspartate-rich DDXXD motif, and 24 phosphorylation sites, suggesting that the KS protein was highly conserved and that the structures of these three domains may be critical for the functionality of KS across different plant species. This finding aligns with previous research conducted on rice, *S. dulcis*, and *P. glauca* [4,16,18]. The results of subcellular localization indicated that the KS gene functions in both the nucleus and cytoplasm, which was consistent with the bioinformatics predictions of bioinformatics, as well as the reports in *Arabidopsis* [28]. The plastid localization of KS was closely related to its function in GA biosynthesis, as the precursor substances of gibberellin were synthesized in plastids [29]. The results of prokaryotic expression were indicated that the KS gene also had the function of enhancing cell resistance to salinity stress. The increase in prokaryotic cell resistance was due to the high expression of heterological *KS* genes, which produce gibberellin-related metabolites that had the function of resisting plant stress [30].

**Figure 7:**
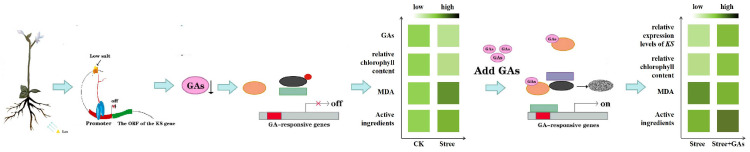
Pattern diagram of *KS* gene expression and GAs accumulation for NaCl stress tolerance from *A. roxburghii*.

The biosynthesis of GAs in plants begins with the activity of KS. Results from qRT-PCR indicate that the expression of the *ArKS* gene was significantly reduced following NaCl treatment. The expression of KS genes was also affected by abiotic stress, in which the expression of KS genes was down-regulated under salt stress in rice [14]. The total amount of GAs was decreased from 16.21 ng/mg to 14.56 ng/mg in *A. roxburghii*. Research has shown a positive correlation between the expression of KS genes and the enrichment of GAs, which has been validated by prokaryotic expression experiments [14]. NaCl stimulation interacts with the promoter of the KS gene, causing a decrease in its expression. At the same time, under NaCl stimulation, plants resist adverse stimuli by consuming GAs, further reducing the content of GAs (Figure 7). GAs were recognized as hormones that play a crucial role in regulating hypocotyl elongation and germination under abiotic stress [31]. The influence of abiotic stress on GA levels occurs through the regulation of the genes, which are crucial enzymes that limit the speed of GA biosynthesis [32]. Different abiotic stresses have varying effects on the expression of key rate-limiting genes in plant GA metabolic pathways [33].

The accumulation of active ingredients from polysaccharides, total flavonoids, and free amino acids was increased during the 15 and 20 days of the inductions, with different ranges between different products. The findings not only clarify the enhanced accumulation of active compounds in *A. roxburghii* under NaCl stress conditions but also validate the positive relationship between GAs and the levels of active ingredients. It was common to increase the content of active ingredients through abiotic stress, but abiotic stress had irreversible effects on plant growth [22]. GAs played a crucial role in regulating plant growth and stress tolerance [26].

## 5. Conclusions

The ORF sequences of the *ArKS* gene were sourced. Through bioinformatics analysis, similarities in *ArKS* proteins and their conserved domains were identified in orthologs across related species. The *ArKS* protein was lacking a kytic vacuole. Transformants exhibited a marked improvement in salt tolerance when subjected to NaCl stress conditions. The *ArKS* genes respond to environmental stimuli and play a key role in the synthesis of active ingredients through the modulation of GAs content in *Anoectochilus*. Additionally, the polysaccharides, total flavonoids, and free amino acids underwent significant changes under salt stress. GAs could not only enhance salt tolerance but also play a key role in the synthesis of active components in *A. roxburghii*.

## Data Availability

The data will be available upon request.

## Supplementary Materials

The following supporting information can be downloaded at https://www.mdpi.com/article/10.3390/genes16080914/s1: Figure S1: The plant of *A. roxburghii*. Figure S2: The fragments of the K*S* gene from cDNA in *A. roxburghii*; Figure S3: The fragments of the K*S* gene from cDNA in *A. roxburghii*; Figure S4: The structure of vector pET-28a(+)-*KS*.
