# Supplementary material for: Overexpression of Ent-Kaurene Synthase Genes Enhances Gibberellic Acid Biosynthesis and Improves Salt Tolerance in Anoectochilus roxburghii (Wall.) Lindl
Source: Genes (Basel). 2025 Jul 30;16(8):914. doi: 10.3390/genes16080914 (PMC12386170; doi:10.3390/genes16080914)
Supplement: Supplementary file 1 [file genes-16-00914-s001.zip › genes-3744811-supplementary.pdf]

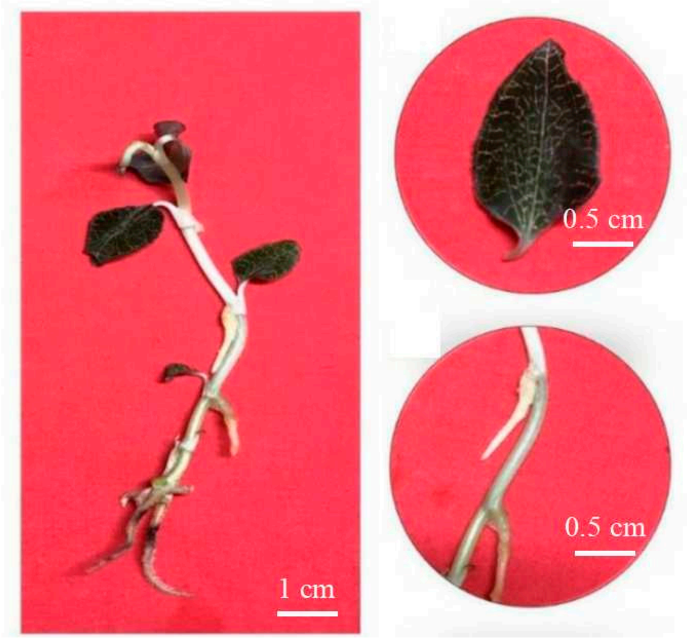

Figure S1. The plant of *A. roxburghii* [21].

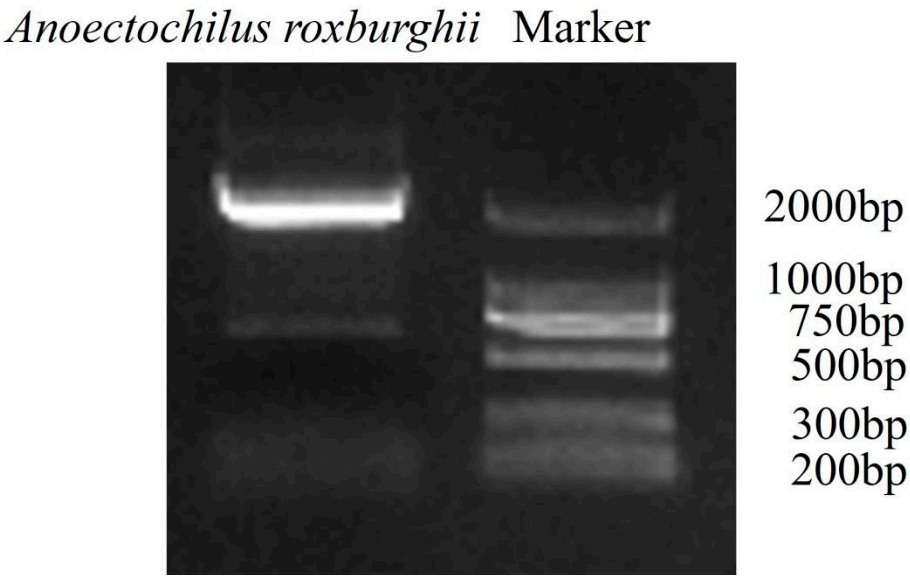

Figure S2. The fragments of the K*S* gene from cDNA in *A. roxburghii*.

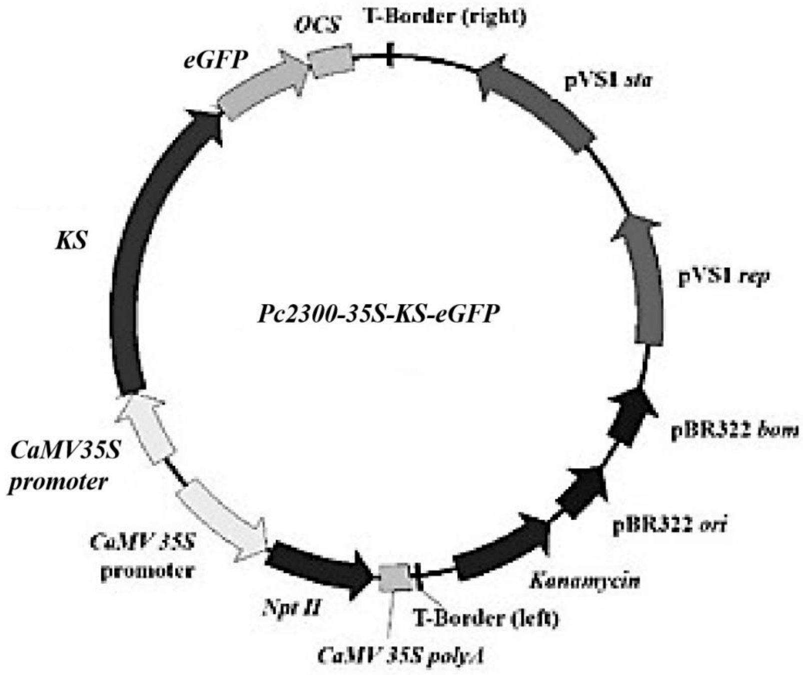

Figure S3. The structure of vector pCAMBIA2300-*ArKS-eGFP*.

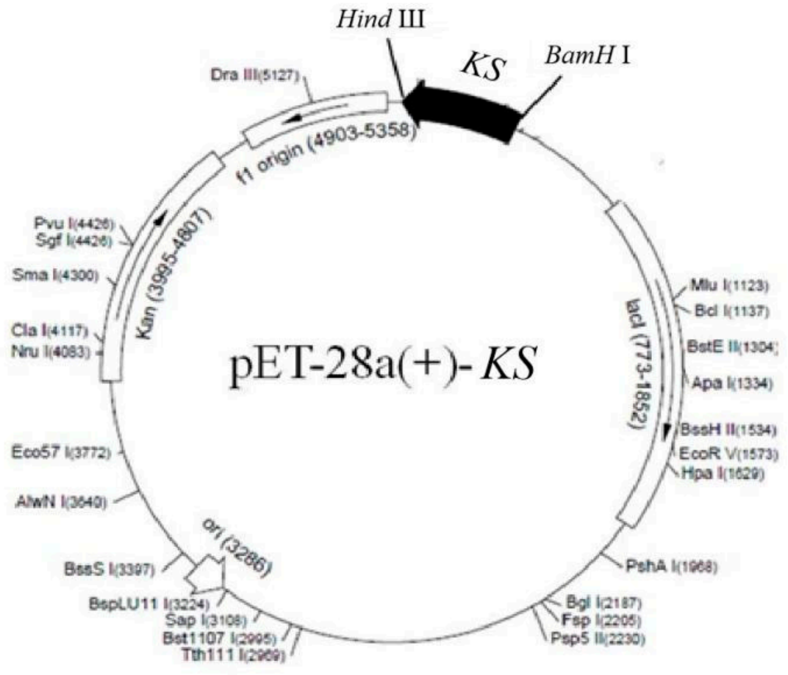

Figure S4. The structure of vector pET-28a(+)-*KS*.
